# Atypical Presentation and Diagnostic Challenges of Appendicitis in an 85-Year-Old Male: A Case Report

**DOI:** 10.7759/cureus.65622

**Published:** 2024-07-29

**Authors:** Thamir Hashim, Yara A AlTahan, Moayad A Elgassim, Hany A Zaki, Mohamed Elgassim, Amro Abdelrahman

**Affiliations:** 1 Emergency Medicine, Hamad Medical Corporation, Doha, QAT; 2 Community Medicine, Hamad Medical Corporation, Doha, QAT; 3 Medical Education, Hamad Medical Corporation, Doha, QAT

**Keywords:** case report, geriatric appendicitis, ruptured appendicitis, negative appendicitis, atypical appendicitis

## Abstract

Acute appendicitis in elderly individuals is uncommon and poses a significant challenge due to atypical symptomatology. An 85-year-old male presented to the emergency department (ED) with abdominal pain associated with nausea and reduced oral intake. Physical examination revealed diffuse abdominal tenderness. He was initially treated for constipation with an enema and discharged. Two days later, the patient returned with worsened pain and a new onset of fever. Examination revealed guarding. Lab results showed significant elevation in C-reactive protein (CRP) and white blood count (WBC). A contrast-enhanced computed tomography (CT) scan showed evidence of a perforated appendix. He was admitted into the surgical ward and improved on conservative treatment.

This case describes an atypical presentation of acute appendicitis in an elderly patient, emphasizing the importance of recognizing unusual presentations in this population. Early use of contrast-enhanced CT scans is crucial for accurate diagnosis and improving patients outcomes.

## Introduction

Acute appendicitis is the most common surgical emergency with a lifetime risk of up to 8% [1]. Appendicitis is commonly seen in younger age groups, but the epidemiology of appendicitis has been changing, with a higher incidence of the disease occurring in the elderly population. This can partly be explained by the increase in life expectancy; however, this observation requires further investigation [2]. Around 5-10% of cases are reported to occur in the elderly population according to literature, however the age for the elderly in the available studies ranges from 60 to 80 years [2]. A study conducted in a hospital covering all appendicitis cases over a 10-year period revealed that only 3% of all cases belonged to the 80-89 age group. Advancing age adversely affects clinical diagnosis, the stage of disease, and the outcome of patients [3]. Perforated appendicitis and septic progression are the main causes of undesirable outcomes [3].

With increasing age, the ability to sense pain decreases as well as other clinical signs may not be very apparent. In addition, serum biomarkers may not be very suggestive of appendicitis; therefore, scoring systems for acute appendicitis, which rely on clinical signs and symptoms and serum inflammatory markers can’t be used in the elderly populations. Due to the broad spectrum of conditions that can occur, it is suggested that all elderly patients presented to the hospital with acute abdominal pain should get a CT done [2]. CT can influence the treatment plan in up to 65% of patients with positive CT findings, medical management in 52%, and surgical management in 48% [2]. It can be said as a result that the early use of CT scans can cut short the way to the appropriate treatment in elderly patients with acute abdominal pain.

Although regarded as a benign disorder, it becomes serious when the appendix perforates. Appendiceal Perforation increases both mortality and morbidity. Due to atypical presentation and delay in making a diagnosis, perforation is more frequently seen in elderly patients [3]. Elderly patients were found to have a higher incidence of complicated appendicitis with reported rates of perforation as high as 70% [4].

## Case presentation

**Figure 1:**
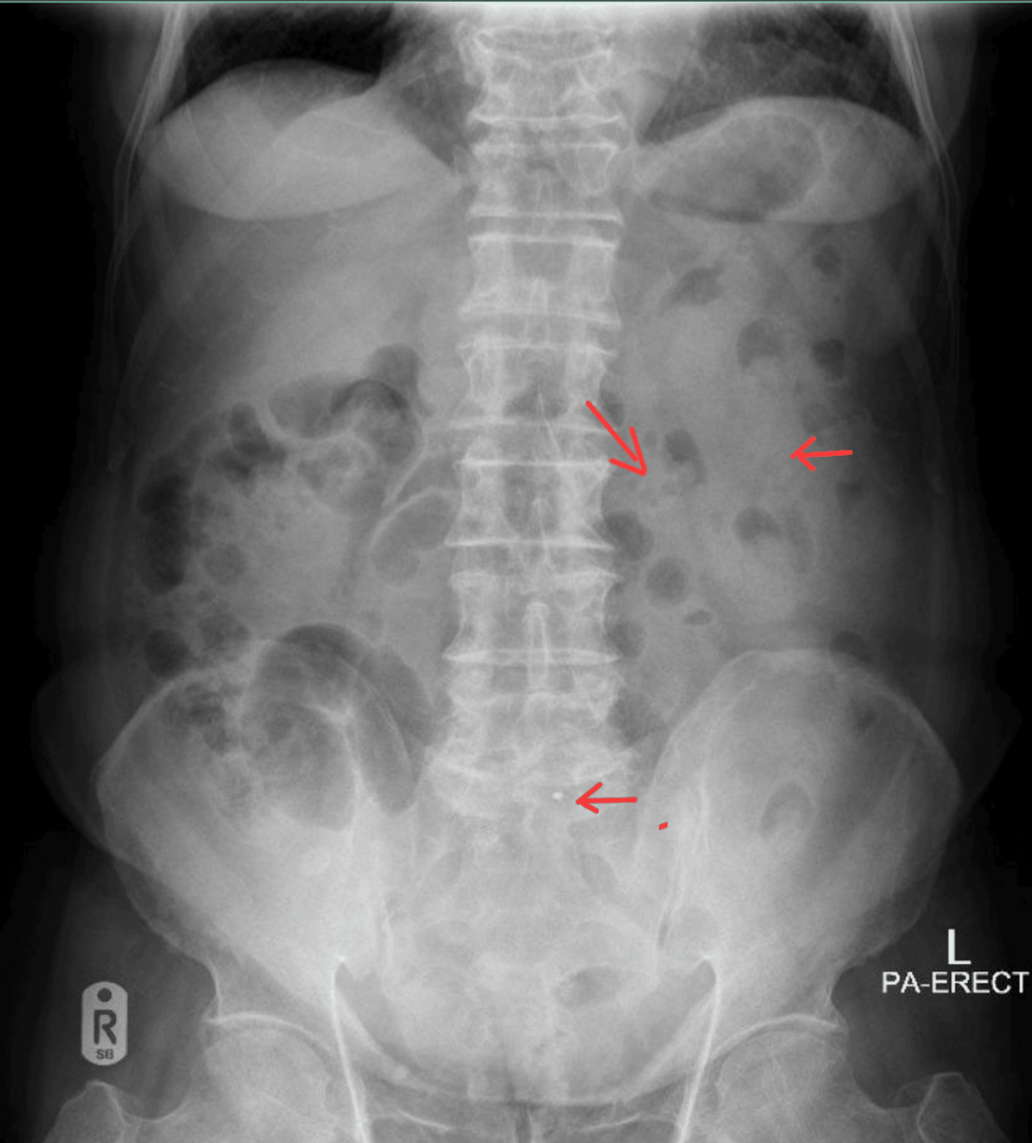
Abdominal X-Ray (XR) Fecal loading in the colon (red arrows).

An 85-year-old male with a known case of hypertension, hyperlipidemia, and prostate cancer on hormone therapy (goserelin injection every three months) presented to the ED with diffuse abdominal pain for four days. The pain was associated with nausea and reduced oral intake, but no change in bowel movements. Physical examination revealed diffuse abdominal tenderness but no rebound tenderness, while vitals were unremarkable. Basic labs, C-reactive protein (CRP), urinalysis, cardiac markers, and abdominal X-Ray (XR) were ordered. Laboratory examination reports were insignificant; however, abdominal XR revealed fecal loading in the colon (Figure 1).

The patient was treated as a case of constipation and given an enema, after which he experienced some pain improvement and was discharged. Two days later, the patient came back to the ED as his pain increased in intensity, and he developed a fever. An abdominal examination revealed guarding and CRP was noticed to have increased from 5 (two days ago) to 276 mg/L and white blood count (WBC) from 6 to 12 (Table 1).

**Table 1:** Laboratory examination results

Test	Result	Normal range
White blood count	12 x10^3^/μL	(4-10) x10^3^/μL
Neutrophil	80%	40-60%
CRP	276 mg/L	<5 mg/L

**Figure 2:**
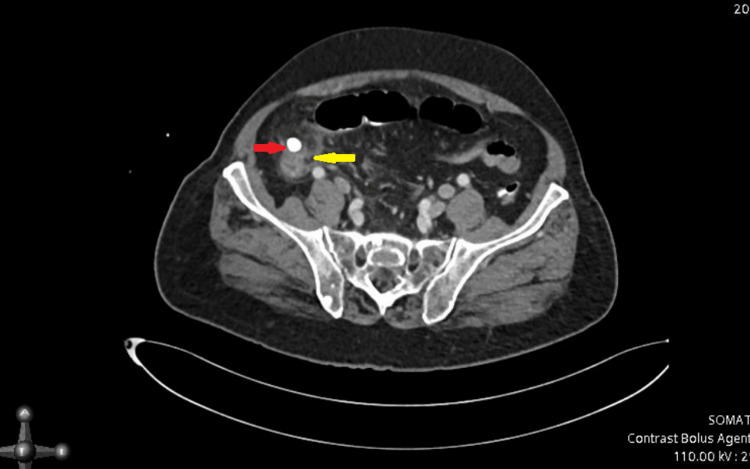
CT abdomen with contrast There is an ill-defined area of pericecal/right paracolic localized focal collection with fat stranding associated with adjacent bowel wall thickening involving the caecal base (yellow arrow). Approximately 10 x 9 mm hyperdense structure seen in the right iliac fossa likely appendicolith (red arrow). The appendix could not be seen separately.

CT abdomen with contrast was requested. A contrast-enhanced CT scan of the abdomen revealed an ill-defined area of pericecal and right paracolic localized focal collection with fat stranding, accompanied by adjacent bowel wall thickening involving the cecal base. Additionally, a hyperdense structure approximately 10 x 9 mm in size was observed in the right iliac fossa, likely representing an appendicolith. The appendix could not be visualized separately. These findings are suggestive of perforated appendicitis (Figure 2).

As a result, the patient was transferred to the surgery ward, where he received conservative management. He was started on ceftriaxone (2 g, thrice a day (TID)) for five days, which was later changed to piperacillin/tazobactam (4.5 g, TID) as per the recommendation of the infectious disease (ID) team. The patient's symptoms started to improve, and he was discharged a week later in stable condition.

## Discussion

Acute appendicitis is predominantly caused by the obstruction of the lumen, resulting in inflammation, ischemia, and, subsequently, perforation, peritonitis, or a contained abscess [5]. The risk of appendicitis perforation in elderly patients includes age, male sex, fever, anorexia, retrocaecal anatomical position of the appendix, left-shift of leucocytes, delay in presentation, and surgical intervention [6]. Typically, symptoms of acute appendicitis include colicky pain that begins in the periubilical region and then becomes a sharp, constant pain. This pain then shifts to the right lower quadrant region. Associated symptoms are anorexia, nausea, vomiting, and fever [5]. Signs of acute appendicitis are McBurney’s sign, rebound tenderness, Rovsing sign, psoas sign, and obturator sign [7].

A review of the literature shows similar cases of appendicitis in the elderly with a wide range of presentations. Most patients had common symptoms such as fever and/or right lower quadrant tenderness [8-10]. Some patients' symptoms deter away from the diagnosis of appendicitis, such as epigastric pain [11] left abdominal pain [10,12,13] thigh pain [14] hematochezia [15].

In elderly patients with appendicitis, the diagnosis may be challenging as the classical symptoms may not be prominent, or the presentation may be non-specific. However, recent updates in the literature have helped aid this process. Biomarkers for appendicitis include total leucocyte count (TLC) and C-reactive protein (CRP), along with recent indicators such as absolute neutrophil count, serum amyloid A (SSA), calprotectin (CP), and myeloid-related protein 8/14 (MRP 8/14O) have shown high sensitivity and negative predictive value. Hyponatremia and hyperbilirubinemia have been shown to predict complicated appendicitis, while the Delta neutrophil index has been examined as a reliable biomarker in the elderly [16]. Because clinical and laboratory parameters alone have low diagnostic accuracy, different scoring systems have been used for the diagnosis and risk stratification of acute appendicitis. 

The Alvarado scoring system was utilized for the diagnosis of acute appendicitis, and it has high sensitivity but low specificity, making it helpful in ruling out acute appendicitis in the triage assessment phase [17]. The preferred imaging technique for the abdomen is contrast-enhanced CT; children and expectant mothers should use magnetic resonance imaging and ultrasound instead. Appendiceal wall thickening or augmentation, peri-appendiceal fat stranding, and/or appendicolith are among the possible findings on CT [18]. In our patient, the final diagnosis was reached through contrast-enhanced CT of the abdomen.

Imaging methods for diagnosing acute appendicitis include ultrasonography (USG), contrast-enhanced CT scan (CECT), and magnetic resonance imaging (MRI). The first-line imaging modality is USG. If USG is unclear and appendicitis is suspected, a low-dose CECT scan is utilized in suspicion of appendicitis, as it is the gold standard [16]. Our patient’s CT scan showed localized focal collection, bowel wall thickening, and a hyperdense structure in the right iliac fossa. Findings were suggestive of a perforated appendix. 

Laparoscopic appendectomy is generally preferred as the first choice when the resources are available. This preference is due to its high first-year and initial treatment success rate compared to non-operative inventions. However, a higher risk of postoperative complications can arise from surgical intervention [19]. Alternatively, non-operative management (NOM) is to avoid surgery through antibiotics [16]. Nonoperative management has shown a 3.72% decrease in complications risk and a 1,82% increase in mortality, as well as longer hospitalization and increased cost [20]. Our patient was treated conservatively with ceftriaxone and later on a piperacillin and tazobactam combination. He was discharged after a week in stable condition.

Study limitations

This report is limited by the patient being lost to follow-up, and thus, we’re unable to provide long-term progress on the patient’s status.

## Conclusions

This case highlights the importance of recognizing atypical presentations of acute appendicitis in the elderly population. Clinicians must look beyond traditional symptoms when assessing abdominal pain in this unique demographic. The patient's initial presentation with non-specific symptoms underscores the need for a broad differential diagnosis. The definitive diagnosis was achieved through the early use of contrast-enhanced CT scans, which proved essential for accurate diagnosis and timely management. This case emphasizes the value of advanced imaging techniques in improving diagnostic accuracy and patient outcomes in elderly individuals with abdominal pain, potentially reducing the risk of complications such as perforation.
